# Breast Cancer-Associated Fibroblasts: Where We Are and Where We Need to Go

**DOI:** 10.3390/cancers8020019

**Published:** 2016-01-27

**Authors:** Rachel J. Buchsbaum, Sun Young Oh

**Affiliations:** 1 Molecular Oncology Research Institute and Department of Medicine, Division of Hematology-Oncology, Tufts Medical Center, Boston, MA 02111, USA; 2 Department of Medicine, Division of Medical Oncology, Montefiore Medical Center, New York, NY 10467, USA; suyoung@montefiore.org

**Keywords:** breast cancer, tumor microenvironment, cancer-associated fibroblasts

## Abstract

Cancers are heterogeneous tissues comprised of multiple components, including tumor cells and microenvironment cells. The tumor microenvironment has a critical role in tumor progression. The tumor microenvironment is comprised of various cell types, including fibroblasts, macrophages and immune cells, as well as extracellular matrix and various cytokines and growth factors. Fibroblasts are the predominant cell type in the tumor microenvironment. However, neither the derivation of tissue-specific cancer-associated fibroblasts nor markers of tissue-specific cancer-associated fibroblasts are well defined. Despite these uncertainties it is increasingly apparent that cancer-associated fibroblasts have a crucial role in tumor progression. In breast cancer, there is evolving evidence showing that breast cancer-associated fibroblasts are actively involved in breast cancer initiation, proliferation, invasion and metastasis. Breast cancer-associated fibroblasts also play a critical role in metabolic reprogramming of the tumor microenvironment and therapy resistance. This review summarizes the current understanding of breast cancer-associated fibroblasts.

## 1. Introduction

Breast cancer is a heterogeneous disease. Clinically, it has been classified based on the expression level of hormone receptors and HER2, a member of the epidermal growth factor receptor family. This classification enables both prognostic and predictive decision-making. More recently, gene expression profiling has enabled classification of breast cancer into molecular subtypes, with greater understanding of disease characteristics and outcomes [1,2,3,4,5]. Cancers develop in complex tissue environments, which they depend on for sustained growth, invasion and metastasis [6]. The cancer microenvironment also has a critical role in tumor initiation, proliferation and metastasis.

The tumor microenvironment is composed of cellular and non-cellular components. Multiple different cell types comprise the cellular compartment of the tumor microenvironment, including immune cells, endothelial cells and fibroblasts. Fibroblasts are the most abundant cell type in tumor-associated stroma, with multiple roles, including deposition of extracellular matrix and basement membrane components, regulation of differentiation events in associated epithelial cells, modulation of immune responses and homeostasis mediation [7,8]. Emerging data have shown that cancer-associated fibroblasts (CAFs) are actively involved in tumor initiation and progression. Here, we will review the multifunctional roles proposed for breast cancer-associated fibroblasts in breast cancer initiation and progression.

## 2. Definition of Breast Cancer-Associated Fibroblasts

While it is increasingly recognized that breast cancer-associated fibroblasts are active players in breast tumor progression, currently there is no single precise definition of CAFs or breast CAFs, due to the absence of uniquely-identifying markers, as well as varying possible cellular origins of CAFs. Cancer-associated fibroblasts have traditionally been defined as α-smooth muscle actin (α-SMA)-positive peritumoral fibroblasts, which are morphologically spindle-shaped cells [9,10]. However, α-SMA expression in fibroblasts is not restricted to cancer-associated or normal fibroblasts and is observed during wound healing and skeletal muscle development in normal fibroconnective disease [9].

### 2.1. There Is No Specific Marker for Breast Cancer-Associated Fibroblasts

Despite the identification of various molecular markers in fibroblasts associated with cancers, none are specific for CAFs. In general, CAFs demonstrate high expression of α-SMA, p53, podoplanin, CD10, fibroblast activation protein (FAP), matrix metalloproteinases (MMPs), tenascin-C and platelet-derived growth factor (PDGFRα/β) and lose caveolin-1 (Cav-1) expression [10,11].

#### 2.1.1. α-Smooth Muscle Actin

Considering tumors as “wounds that do not heal”, fibroblasts in the tumor microenvironment share similarities with fibroblasts that are involved in wound healing, including α-SMA expression [12,13]. Myofibroblasts, mesenchymal cells sharing features of fibroblastic and smooth muscle cell, are morphologically defined as large spindle-shaped cells with indented nuclei, reflecting cellular contraction [9]. Immunocytochemical characterization indicates that α-SMA, one of six actin isoforms predominant in vascular smooth muscle, is the most significant marker of myofibroblast differentiation. These myofibroblasts were initially described in wound-healing granulation tissues and are also observed in the tumor stroma of various types of cancers. Studies have shown that increased expression of α-SMA in breast cancer stroma is associated with higher histologic grade, lymph node metastasis, microvessel density and poor prognosis [14,15,16,17].

#### 2.1.2. Caveolin-1

Caveolae are sphingolipid and cholesterol-rich invaginations of the plasma membrane in which caveolins are the principal membrane components. Cav-1 is a principle caveolin that acts as a scaffold protein to sequester and organize multi-molecular signaling complexes involved in diverse cellular activities, such as lipid transport, membrane trafficking, gene regulation and signal transduction [18]. Ablation or mutation of Cav-1 is one of the features of fibroblasts in tumor tissues [14]. Recent data showed that the loss of stromal Cav-1 is associated with advanced breast tumor stage, increased lymph node metastasis, decreased disease-free survival or overall survival, higher histologic grade and greater resistance to endocrine therapy [19,20,21,22,23,24,25]. The loss of stromal Cav-1, rather than epithelial Cav-1 expression, was an independent prognostic factor in breast cancer outcome [24,25]. However, the role of Cav-1 in the molecular cross-talk between tumor and stroma is not yet well characterized [21].

#### 2.1.3. Fibroblast Activation Protein/Seprase

FAP is an *N*-glycosylated, type II integral membrane protein that belongs to the family of plasma membrane-bound serine proteinases [26]. It is expressed in reactive stromal fibroblasts in epithelial cancers, granulation tissue in healing wounds and some fetal mesenchymal tissues, but not in adult normal tissue. In invasive ductal breast carcinomas, the expression of FAP was restricted to the stromal cells, and increased FAP expression was an independent prognostic factor associated with better prognosis [26].

#### 2.1.4. Tenascin-C

Tenascin is an extracellular glycoprotein produced by stromal fibroblasts and epithelial cells of normal and malignant breast tissue [27]. Its expression is associated with poor prognosis in breast cancer patients [27,28,29]. In one study of 134 breast cancer cases comparing the prognostic value of various extracellular matrix components, including tenascin, fibronectin, collagen type IV and laminin, only tenascin expression was an independent prognostic factor. Stromal tenascin expression was inversely correlated with breast cancer prognosis [27]. Another study showed that breast tumors with increased stromal tenascin expression exhibited increased frequency of lymph node metastasis and decreased overall survival [28].

#### 2.1.5. Podoplanin

Podoplanin is a mucin-type glycoprotein known to be a marker for lymphangiogenesis, as it is highly expressed in the endothelium of lymphatic vessel, rather than blood vessels [30]. While the precise biological function of podoplanin in normal tissues remains to be elucidated, its expression in various cancers has been demonstrated [31]. In breast cancer, podoplanin expression by CAFs was associated with increased breast tumor size, histologic grade, lymph node metastasis, lymphovascular invasion and worse disease-free and overall survival [32,33,34,35].

#### 2.1.6. Platelet-Derived Growth Receptor α/β

Platelet-derived growth factor receptors exert important control functions in mesenchymal cells, including pericytes, fibroblasts and vascular smooth muscle cells, during development. PDGFR overactivity has been linked to tumorigenesis [36]. Expression of PDGFR in breast tumor stroma was significantly associated with high histopathologic grade, estrogen receptor negativity, high expression of HER2 receptor, shorter disease-free survival and overall survival [37,38]. One study examined PDGFR α/β expression and disease outcome in 45 patients with breast cancer who relapsed after aromatase inhibitor treatment [38]. Breast tumor and stromal expression of PDGFR α/β was significantly increased during the development of resistance to aromatase inhibitor treatment.

Of the markers known to be expressed or altered in CAFs, none are specifically altered in breast CAFs. Neither a meta-analysis of the correlation between biomarkers of breast CAFs and regional metastasis [39] nor the study of an orthotopic mouse xenograft model of breast cancer [40] identified specific biomarkers that can be used to identify breast CAFs. It is likely that breast CAFs themselves are heterogeneous, analogous to the heterogeneity in epithelial breast cancers themselves. A better definition of breast CAF sub-types would facilitate understanding of breast CAF biology.

### 2.2. Human Breast Cancer-Associated Fibroblasts Are Genetically Different from Normal Fibroblasts

Gene expression profiling has provided molecular subtypes of breast cancer based on the analysis of epithelial tumor cells. With growing evidence of the critical contribution of the tumor microenvironment in cancer development, several studies have investigated the gene expression profile of breast CAFs. When compared to fibroblasts from women with benign breast disorders, the gene expression pattern of stromal fibroblasts derived from malignant breast tissue of women with invasive breast cancer showed a distinctive gene expression pattern that differed from normal breast fibroblasts [41]. In that study, the majority of upregulated genes in the breast CAFs encoded tumor-promoting cytokines, transcription factors and cell matrix-associated proteins. Another study compared the gene expression profiles in paired breast cancer-associated fibroblasts and normal fibroblasts from six primary human breast carcinoma specimens [42]. Twenty-one genes known to be involved in paracrine or intracellular signaling, transcriptional regulation, extracellular matrix and cell adhesion/migration were upregulated in the breast CAFs, while ten genes involved in steroid hormone metabolism and polycyclic aromatic hydrocarbon detoxification were downregulated in breast CAFs. Of note, the estimated probability of the gene expression variance of normal breast fibroblasts was higher than that of breast cancer-associated fibroblasts in that study. This suggests underlying heterogeneity among normal stromal fibroblasts, as well. A third study compared the gene expression profile of primary breast CAFs from different subtypes of breast cancers [43]. Though the morphology was similar between CAFs from different sub-types, the molecular profiles of these breast CAFs varied depending on the original breast cancer subtype. The genes involved in cytoskeleton and integrin signaling pathways were significantly upregulated in breast CAFs isolated from HER2-overexpressing breast cancers when compared to those isolated from triple-negative breast cancers or estrogen receptor-positive breast tumors. Functionally, breast cancer-associated fibroblasts derived from HER2+ breast cancers significantly enhanced the transwell migration of the T47D breast cancer cell line, as compared to the fibroblasts isolated from ER+ or triple-negative breast cancers. This suggests that CAFs may be specifically educated by the associated breast cancer cells. In another study, transcriptional effects of 1α,25 dihydroxyvitamin D3 on breast CAFs were different from the effects on paired normal fibroblasts [44]. In breast CAFs, 1α,25 dihydroxyvitamin D3 decreased the expression of genes associated with proliferation (NRG1, WNT5A, PDGFC) and increased the expression of genes involved in immune modulation (NFKB1A, TREM-1). On the other hand, 1α,25 dihydroxyvitamin D3 induced genes involved in anti-apoptosis, detoxification, the antibacterial defense system and protection against oxidative stress in normal fibroblasts.

A study utilizing primary cancer cells injected into mouse mammary glands humanized with primary breast fibroblasts compared the effects of fibroblasts from Caucasian (Cau) and African American (AA) patients, as well as whole breast extracellular matrix molecules (ECM) [45]. Mass spectrometry demonstrated only limited overlap (approximately 22%) in ECM proteins from the two populations. ER+/PR+ cells were more aggressive when exposed to Cau ECM, while ER-/PR- cells were more aggressive when exposed to AA ECM.

Recent gene expression profiling studies have also demonstrated the impact of tumor-associated stroma on breast cancer outcomes [46,47]. Breast cancer prognosis estimated by stroma-derived prognostic predictor, which was developed from gene profiling of 53 primary breast tumor stroma, was independent of estrogen receptor expression, HER2 expression, grade, lymph nodal involvement, age, previous chemotherapy or hormonal therapy status [47]. Gene expression profiling of extracellular matrix enabled sub-classification into subsets that correlated with outcome among early-stage breast cancer [46].

Epigenetic alterations have also been observed in breast CAFs [48,49,50]. The novel method of methylation-specific digital karyotyping demonstrated epigenetic alterations in stromal fibroblasts, as well as epithelial and myoepithelial cells in normal breast tissues compared to *in situ* and invasive carcinomas [49]. Another study demonstrated that DNA methylation profiles derived from 143 human breast tumors showed significant differences in both HER2 expression and DNA methylation of five genes. Three of these five genes were also methylated in breast tumor stroma, as well as the cancer cells [48]. Recently, it was reported that normal breast fibroblasts co-cultured with breast cancer cells increasingly promote cancer cell invasion, in part through upregulation of ADAM metallopeptidase with thrombospondin type 1 motif, 1 (ADAMTS1) due to decreased binding of the histone methyltransferase EZH2 to the ADAMTS1 promoter [50].

There are some caveats to drawing over-arching conclusions from the profiling studies. Though it appears that the gene expression profiles of breast CAFs are different from those of their normal counterparts and that their expression patterns vary depending on the breast cancer subtypes, most gene profiling reports involve a relatively small size of samples. In addition, fibroblast gene expression may be altered during *in vitro* passage after isolation from primary tissues. Currently, it is not clear whether the genetic and epigenetic alterations of breast CAFs are the prerequisite or consequence of the breast cancer initiation and progression. Analysis of larger sample sizes with varying breast cancer subtypes and patient clinical characteristics, including different ethnicities, will be needed to provide complete understanding of the genetics of breast CAFs.

### 2.3. Origin of Breast Cancer-Associated Fibroblasts Is Controversial

Despite being the most prevalent cell type in the tumor microenvironment, the origin of breast CAFs has not been conclusively determined. There is varying evidence supporting origins of breast CAFs from resident fibroblasts, bone marrow-derived mesenchymal stem cells or cancer cells that undergo epithelial- or endothelial-mesenchymal transition.

#### 2.3.1. Breast Cancer-Associated Fibroblasts May Originate from Resident Fibroblasts

To investigate the origin of breast cancer-associated fibroblast, three different types of stromal cells (fibroblasts, vascular smooth muscle cells and pericytes) were isolated from primary breast tissue samples and co-cultured with tumor cells [51]. Fibroblasts readily converted into a graded pattern of myogenic differentiation and demonstrated increased expression of α-smooth muscle. In contrast, vascular smooth muscle cells (VSMCs) and pericytes did not change appreciably. Progressive conversion of resident human mammary fibroblasts into CAFs was shown in a co-implantation breast tumor xenograft model [52]. These breast CAFs acquired autocrine signaling loops mediated by TGF-β and SDF-1, which induce and maintain differentiation of fibroblasts into myofibroblasts, promoting tumor progression.

#### 2.3.2. Breast Cancer-Associated Fibroblasts May Originate from Mesenchymal Stem Cells

Prolonged exposure (30 days) of human bone marrow-derived mesenchymal stem cells to conditioned media from MDA-MB-231 breast cancer cells resulted in myofibroblast differentiation, characterized by high expression of α-SMA, vimentin, fibroblast surface protein (FSP) and SDF-1 [53]. The gene expression profile of human bone marrow-derived mesenchymal stem cells treated with MDA-MB-231 conditioned media was similar to that reported for CAFs. A recent report further delineated the possible mechanism of transformation of mesenchymal stem cells into breast CAFs. The phosphoglycoprotein osteopontin induces the transformation of mesenchymal stem cells into CAFs, mediated by activation of transcription factor, myeloid zinc finger 1 (MZF1) and induction of mesenchymal stem cell production of TGF-β [54].

#### 2.3.3. Breast Cancer-Associated Fibroblasts May Originate from Epithelial Cells or Endothelial Cells through Epithelial-to-Mesenchymal Transition or Endothelial-to-Mesenchymal Transition, Respectively

Finally, one group has shown that EMT or endothelia-to-mesenchymal transition (EndMT) is not only a crucial step during cancer progression, but also that these processes could produce CAFs [7,55,56]. While these results were shown in non-breast cancer systems, they may be applicable to the origin of breast CAFs, as well.

Breast CAFs are a critical cellular component of the breast tumor microenvironment. Despite significant effort to delineate specific markers, genetic profiles and the origin of breast CAFs, conclusive and unique definitions of breast cancer-associated fibroblasts are lacking at present, likely due to CAF heterogeneity and relatively small sample sizes used in individual studies. Studies with larger sample sizes in association with different subtypes of breast cancer, as well as host characteristics would likely provide better understanding of breast CAFs and their biology.

## 3. Role of Breast Cancer-Associated Fibroblasts in Breast Tumor Initiation and Growth

Under normal physiological conditions, stroma serves as an important barrier to epithelial cell transformation, and the interplay between epithelial cells and the microenvironment maintains epithelial polarity and modulates growth inhibition [6]. However, the stromal compartment undergoes changes in association with emerging epithelial lesions and has a key role in cancer initiation and progression. There is developing evidence for different roles of fibroblasts in normal tissues compared to cancer tissues.

### 3.1. Effects of Normal Fibroblasts on Tumor Progression

A study investigating the ability of normal fibroblast to induce primary breast carcinoma cell reversion, defined by the formation of an apico-basally-polarized acinus-like structure of primary breast carcinoma cells with lumen formation, in a three-dimensional collagen-I gel co-culture system, showed that the presence of normal mammary fibroblasts reverted primary breast cancer cells morphologically [57]. However, primary breast cancer cell growth was not significantly altered by the presence of normal mammary fibroblasts. In this study, the effect of normal mammary fibroblasts on the primary breast cancer cells was not compared to that of cancer-associated fibroblasts. The influence of normal and tumor associated fibroblasts on co-cultured normal (MCF10A) and preneoplastic breast cell lines (MCF10AT1-EIII8) was compared in a Matrigel three-dimensional (3D) model [58]. Whereas the normal mammary fibroblasts inhibit or retard morphological conversion and growth of MCF10A or MCF10AT1-III8 cells, fibroblasts derived from breast tumors induced epithelial cell growth and ductal-alveolar morphogenesis. In this study, only fibroblasts from breast tissue, not from other tissues, induced epithelial cell growth and morphogenesis. A different group found that fibroblasts from both normal breast tissue and breast cancer tissue suppressed proliferation of MCF10A cells, but only normal fibroblasts inhibited proliferation of the more transformed MCF10AT epithelial cell line [59]. Another study showed that both premalignant (human mammary epithelial cells expressing oncogenic Ras) and malignant (MDA-MB-231) breast epithelial cells assume aligned mesenchymal morphology when co-cultured with breast cancer-associated fibroblasts, but not with normal mammary fibroblasts [60]. This mesenchymal phenotype was governed by the extracellular matrix deposition by CAFs when there was direct epithelial cell and fibroblast contact. In an orthotopic mouse model, CAFs increased breast cancer metastasis, while normal fibroblasts reduced tumor growth and metastasis [60]. On the other hand, a xenograft model of human ductal carcinoma *in situ* using the MCF10ADCIS cell line (MCF10ADCIS.com line, a derivative of the MCF10A line that forms DCIS-like lesions) showed that the spontaneous progression from *in situ* to invasive breast carcinoma was enhanced by co-implantation with normal mammary fibroblasts and suppressed by co-implantation with myoepithelial cells that normally line mammary ducts and alveoli [61]. Subsequent study showed that increased invasion of MCF10ADCIS was at least in part due to increased COX-2 expression in tumor epithelial cells provoked by interaction with fibroblasts, and upregulation of COX-2 resulted in increased VEGF and MMP14 expression [62]. Finally, as noted above, studies in a mouse xenograft model found different effects of extracellular matrix (ECM) from premenopausal women on breast cancer cell metastasis depending on ethnicity [45]. Mass spectrometry analysis of ECM showed that ECM proteins from the African American group were primarily related to tumorigenesis, while ECM proteins from the Caucasian group were associated with growth and metastasis.

Normal fibroblasts studied in breast cancer biology are either primary cells isolated from a mammary reduction specimen or immortalized cell lines derived from normal mammary tissue. Considering the heterogeneity of normal fibroblasts, as well as the lack of specific markers differentiating normal from cancerous fibroblasts, it is possible that some of the normal fibroblasts that were used in these studies may have undergone some degree of genetic alteration. In addition, different sub-types of normal breast stromal fibroblasts may exert distinct effects on the associated breast epithelial cells. Currently, the available data are not conclusive as to whether normal mammary fibroblasts suppress or promote breast cancers.

### 3.2. Breast Cancer-Associated Fibroblasts Promote Breast Cancer Initiation and Proliferation

Breast cancer-associated fibroblasts have been shown to promote tumor progression of premalignant and malignant breast epithelial cells *in vivo* and *in vitro* [58,60,63]. Cancer-associated fibroblasts are well-established as a source for growth factors and cytokines known to have critical roles in cancer progression.

#### 3.2.1. Cancer-Associated Fibroblasts Promote Breast Cancer Proliferation by Secreting Various Growth Factors

FGFs, HGF, TGF-β and SDF-1 are reported to be produced by breast cancer-associated fibroblasts and promote tumor proliferation [50,64,65,66,67,68]. FGF7 is a potent growth factor for mammary cells, is produced by breast stromal fibroblasts and induces proliferation of breast epithelial cells [67]. Age is an important risk factor in many cancers, including breast cancer, and cellular senescence is thought to contribute to the effects of aging on cancer development [64]. With induction of breast fibroblast aging through *in vitro* passage in tissue culture, FGF1, plasminogen activator inhibitor-1 and MMP-2 were all increased [66]. HGF, a mediator of cancer development and progression, is mainly secreted from fibroblasts, whereas its receptor, c-Met, is primarily expressed in epithelial cancer cells [69]. In a humanized orthotopic xenograft mouse model, HGF overexpression resulted in increased branching of mammary ducts and hyperproliferation of mammary epithelium [65]. Breast CAFs expressed higher levels of HGF compared to normal fibroblasts, and deprivation of HGF reduced CAF-mediated colony formation of human breast cancer cells [50]. When normal fibroblasts were co-cultured with MDA-MB-468 breast cancer cells, HGF secretion increased colony formation and tumor growth in a mouse model. Heat shock factor 1 (HSF1), a ubiquitously-expressed transcription factor, was also shown to be activated in breast CAFs, and loss of HSF1 in fibroblasts reduced xenograft breast tumor growth, as well as fibroblast production of TGF-β and SDF1 [68].

#### 3.2.2. Cancer-Associated Fibroblasts Promote Breast Cancer Proliferation by Secreting Various Cytokines

SDF-1 (also known as CXCL12) is involved in angiogenesis and direct tumor growth. In a mouse xenograft model, co-implantation of breast CAFs promoted breast cancer cell growth through the secretion of SDF-1 [70]. Another study showed that the obesity cytokine leptin is expressed in primary breast CAFs and promotes breast cancer cell proliferation [71]. A third study demonstrated that expression of IL-6 is approximately 100-fold higher in cancer-associated fibroblasts compared to normal fibroblasts, suggesting that IL-6 may potentiate breast cancer invasiveness [72]. About 70% of breast cancers express the estrogen receptor and are sensitive to estrogen depletion. Conditioned media from breast CAFs enhanced MCF-7 breast cancer cell growth. The activity of 17 beta-estradiol dehydrogenase (E2DH), which reduces estrone (E1) to estradiol (E2), was significantly increased in the media from fibroblasts derived from malignant breast tissue compared to media from normal or benign breast tumor tissue [73], suggesting that CAFs promote breast cancer progression by providing estrogen.

Taken together, breast cancer-associated fibroblasts support breast cancer cell proliferation through secretion of various growth factors and cytokines. However, the mechanisms underlying the upregulation of these factors, the details of cross-talk pathways between factors, as well as downstream pathways are yet to be elucidated. More fundamentally, it is not yet clear whether these breast CAF-secreted factors are the consequence of the cancer effect on the CAFs themselves or initiated before cancer transformation.

Moreover, while much of the available data have shown tumor-promoting effects of breast CAFs on breast cancer cells, note should be made of reports demonstrating cancer-suppressive effects of CAFs, including breast CAFs. Chang *et al*. demonstrated that an interaction between the Robo1 receptor on cancer cells and the ligand Slit2, secreted by fibroblasts, blocks β-catenin nuclear translocation and downregulates c-myc and cyclin D1 [74]. High cancer cell expression of Robo1 correlates with increased survival, while low fibroblast Slit2 expression correlates with lymph node involvement. Xu *et al.* reported that co-implantation of SUM1315 breast cancer cells with immortalized mammary fibroblasts delayed primary tumor formation and decreased lung metastasis [75]. In a murine model of colitis-associated carcinoma, tissue-restricted deletion of fibroblast Iκκβ led to upregulation of the TGFβ genetic signature and promotion of colonic tumor growth, suggesting a tumor-suppressive function of CAF Iκκβ dependent on the release of hepatocyte growth factor [76]. The balance between tumor-promoting and tumor-suppressive effects of CAFs may hinge on heterogeneity across CAF populations, as well as within other components of the tumor microenvironment itself.

## 4. Role of Breast Cancer-Associated Fibroblasts in Tumor Invasion and Metastasis

Cancer metastasis accounts for more than 90% of cancer-related mortality and is largely incurable. Metastasis has been viewed as a linear process involving: (1) local invasion by primary tumor cells through epithelial-mesenchymal transition and extracellular matrix remodeling; (2) intravasation into blood vessel lumina and survival through transport in the vasculature; (3) extravasation into the parenchyma of distant tissues; and (4) formation of micrometastases, with reinitiation of proliferative programs at metastatic sites, which ultimately generates macroscopic, clinically-detectable neoplastic growths at the metastatic site [77]. Another view of metastasis is a parallel progression model, in which tumor cells disseminate very early in malignant progression, colonizing multiple secondary sites at different times and accumulating genetic changes independently of the primary tumor [78]. Multiple studies have investigated the effects of the tumor microenvironment in the metastatic process, showing that cancer-associated fibroblasts may affect multiple steps along the way.

### 4.1. Breast Cancer-Associated Fibroblasts Induce Local Invasion through Epithelial-Mesenchymal Transition and Extracellular Matrix Remodeling

EMT has been described as a cell-biological program that is required for the remodeling of cells and tissue during embryogenesis, wound healing and acquisition of malignant traits by cancer cells [79,80]. Recent data suggest that EMT is not only a mechanism for the initiation of invasion, but also provides stem cell-like properties, such as self-renewal, to breast epithelial cells [81]. Several studies have demonstrated that breast cancer-associated fibroblasts induce EMT in breast cancer cells [60,82,83,84]. Conditioned media from CAFs [84], an indirect co-culture system with CAFs and cancer cells [83] or a direct two-dimensional culture [60] induce EMT of breast cancer cells. One study compared the effects of fibroblasts from the tumor burden zones (CAFs), the distal normal zones (NFs) and the interface zones (INFs) on the breast cancer cells [82]. INFs grew fastest and expressed a higher level of FAP than did NFs or CAFs. In addition, INFs more effectively induced EMT of associated breast cancer cells in a direct co-culture system compared to NFs or CAFs. The authors of this study concluded that fibroblasts from the interface zone of the tumor possess a greater capacity to modulate human breast cancer cells than do NFs or CAFs.

Breast CAFs affect surrounding extracellular matrix (ECM), as well as surrounding cells. ECM deposited by normal fibroblasts exhibits a random mesh-like appearance, but ECM deposited by CAFs is aligned in a parallel pattern [60]. Premalignant breast cancer cells (human mammary epithelial cells expressing oncogenic Ha-Ras) seeded on CAF-deposited ECM acquired a mesenchymal morphology. In this study, protein analysis revealed that ECM deposited by CAFs contains higher level of fibronectin, biglycan and lysyl oxidase, an enzyme involved in ECM remodeling. Recent work demonstrated that miR-200s, a micro-RNA family known to inhibit cell malignant transformation and prevent tumor initiation [85], is downregulated in breast CAFs [86]. Downregulation of miR-200s in normal fibroblasts resulted in increased expression of α-SMA, a marker of fibroblast activation. In addition, a decreased miR-200s level induced expression of Fli-1 (friend leukemia integration 1) and TCF-12 (transcription factor 12), which are correlated with poor outcome in breast cancer patients. In this study, downregulation of miR-200s increased ECM strength and stiffness, dependent on both Fli-1 and TCF12.

In contrast to normal mammary fibroblasts, cancer-associated fibroblasts promote dissemination of premalignant mammary epithelial cells and metastasis of malignant epithelial cells [60]. Cellular senescence is associated with aging and characterized by permanent growth arrest and resistance to apoptosis [87,88]. Several studies have demonstrated that senescent fibroblasts contribute to tumor development [88,89,90]. In a 3D co-culture model, mammary fibroblasts undergoing stress-induced senescence promoted increased migration and invasion of co-cultured mammary epithelial cells, dependent on senescence-induced changes in fibroblast expression of the Rac exchange factor Tiam1 and the integrin-binding phosphoglycoprotein osteopontin [91].

### 4.2. Breast Cancer-Associated Fibroblasts Promote Cancer Cell Transmigration and Metastatic Tropism

In contrast to the multiple studies noted above on the effects of breast cancer-associated fibroblasts on cancer cell migration and invasion, the effects on intravasation and extravasation of cancer cells during metastasis are less understood. However, one study showed that primary breast cancer-associated fibroblasts enhanced MDA-MB-231 breast cancer cell adhesion to human brain microvascular endothelial cells, blood brain barrier (BBB) permeability and disruption and BBB transmigration in a 3D culture model [92].

Despite the theoretical ability of cancer cells to disseminate to a wide variety of secondary loci, preferred metastatic sites are dependent on individual carcinoma types [93]. An unresolved question is whether this tissue tropism reflects a passive process of cancer cell arrest within capillary beds due to geometric constraints of various vascular beds or active homing of cancer cells to specific organs [77]. Recent work suggests that breast cancer-associated fibroblasts may influence the secondary site for the breast cancer cells [94]. Triple-negative breast cancer has a propensity to metastasize in visceral organs. However, triple negative breast cancer cells grown in media supplemented with CXCL12 and IGF derived from CAFs demonstrated increased skeletal metastasis. In addition, CAFs may co-travel with cancer cells in the vasculature, supporting the viability of circulating metastatic cancer cells [95]. In this study, brain metastases from various human cancers, including lung, breast, kidney and endometrium, contained CAFs, defined as α-SMA-expressing spindle stromal cells, which are normally not detected in primary brain tumors or normal brain tissues.

Thus emerging data indicate that breast cancer-associated fibroblasts are actively involved in many of the early steps of breast cancer metastasis.

## 5. Breast Cancer-Associated Fibroblasts Interact with Other Microenvironment Cells in Promoting Metastasis

### 5.1. Angiogenesis and Lymphangiogenesis

Angiogenesis is critical during tumor progression, as cancer cell proliferation and metastasis depend on an adequate supply of oxygen and nutrients and the removal of waste products [96]. In a co-implantation mouse xenograft model with MCF-7 and human fibroblasts, cancer-associated fibroblasts promoted tumor growth and angiogenesis by recruiting endothelial progenitor cells, mediated in part by SDF-1/CXCL12 [70]. Syndecan-1 is a transmembrane cell surface heparin sulfate proteoglycan that acts as an extracellular matrix receptor and participates in cell proliferation, cell migration, cell-cell adhesion, cell matrix interactions and morphogenesis [97]. Stromal fibroblast expression of syndecan-1 enhanced MDA-MB-231 breast cancer cell growth and angiogenesis *in vitro* and *in vivo* [98,99]. Similarly, lymph node metastasis is greatly facilitated by lymphangiogenesis, a process that generates new lymphatic vessels from pre-existing lymphatics or lymphatic progenitors [100,101,102]. Compared to blood vessels, lymphatic vessels offer many advantages for invasion and transport of pre-metastatic cells, including discontinuous basement membrane and loose cell-cell junctions, lower flow rates that increase survival by minimizing shear stress and a 1000-fold higher lymph concentration of hyaluronic acid, a molecule with potent cell-protecting and pro-survival properties [103]. Clinically, increased lymphatic vessel density in breast tumors is associated with lymphatic metastasis and decreased survival [103]. Hyaluronic acid is a major extracellular matrix component that provides tissue hydration and turgidity and also activates intracellular signals through interaction with cell surface receptors [104,105]. In a co-implantation mouse xenograft model, cancer-associated fibroblast from hyaluronic acid-overproducing mouse mammary tumors increased mammary tumor growth and lymphangiogenesis [106]. In another study of 156 invasive ductal breast cancer samples, podoplanin expression in stromal breast cancer-associated fibroblasts was associated with higher grade and triple-negative breast cancer [32]. Eliminating CAFs in a 4T1 murine breast cancer model of metastatic breast cancer by injecting a DNA vaccine targeting FAP led to decreased expression of pro-angiogenic factors, such as VEGF, PDGFR and GM-CSF, and resulted in suppression of angiogenesis and lymphangiogenesis [107]. Taken together, these findings suggest that breast cancer-associated fibroblasts support tumor growth and invasion in part by enhancing angiogenesis and lymphangiogenesis.

### 5.2. Immune System Response

Inflammation is a critical component of tumor progression, and the tumor microenvironment plays a role in promoting chronic inflammation at the tumor site. Chronic inflammation triggers the release of pro-inflammatory cytokines that disrupt the normal cytokine balance and promote tumor cell growth through stimulating angiogenesis and lymphangiogenesis, while also inhibiting activation of cytotoxic immune cells [108]. Cancer-associated fibroblasts secrete high levels of pro-inflammatory cytokines, including IL-1β, IL-8, IL-10, tumor necrosis factor-alpha (TNFα), monocyte chemoattractant protein-1 (MCP-1/CCL2), SDF-1/CXCL12 and interferon-beta (IFN-β), which have a range of effects on the immune system [7]. Cancer-associated fibroblasts from different tumors, including skin, mammary adenocarcinoma in an MMTV-PyT transgenic mouse mammary tumor model [109] and a pancreatic cancer mouse model, exhibited increased expression of a pro-inflammatory gene signature [110].

In the 4T1 murine model of metastatic breast cancer, elimination of CAFs with the anti-FAP DNA vaccine noted above enhanced the antitumor effect of doxorubicin, with decreased primary tumor growth and metastasis [107]. This was associated with both increased IL-1 and IL-7, Th1 cytokines (which favor anti-tumor cytotoxic T lymphocytes), as well as enhanced doxorubicin-induced reduction of Th2 cytokines (which prevent tumor rejection and promote tumor growth). Elimination of FAP-positive fibroblasts also reduced the recruitment of tumor-associated macrophages or myeloid-derived suppressor cells (which produce Th2 cytokines and increase recruitment of dendritic cells and CD8+ T cells). However, another *in vitro* study showed that blood monocytes, precursor cells to tumor-associated macrophages, infiltrated into breast cancer-associated fibroblast spheroids to a greater extent than normal fibroblast spheroids [111]. A different study showed that heterozygous deletion of TGF-β in stromal fibroblasts enhanced distant metastasis and increased inflammatory cytokines, including CXCL12 and CCL2 [112]. Finally, osteopontin is involved in multiple pathophysiologic processes in cancer progression [113]. Murine breast cancer cell-derived osteopontin activated primary normal mammary fibroblasts and increased pro-inflammatory factors, such as CXCL1, CXCL2, IL-6 and COX-2 [114]. Thus breast cancer-associated fibroblasts play a key role in orchestrating chronic inflammation in the tumor microenvironment through modulation of several inflammatory cytokines.

## 6. Breast Cancer-Associated Fibroblasts Contribute to Metabolic Reprogramming of the Tumor Microenvironment

Under normal aerobic conditions, cells utilize glucose to produce pyruvate, which then enters the tricarboxylic acid (TCA) cycle to produce ATP, a process called oxidative phosphorylation. Cancer cells produce energy via the conversion of glucose into lactate, despite the presence of oxygen, a process known as aerobic glycolysis, the “Warburg effect” [115]. A proposed rationale for this metabolic shift is that rapidly-proliferating cancer cells require macromolecules, such as nucleotides, amino acids and lipids, as well as energy. Autophagy is a survival pathway involving degradation of cytoplasmic constituents, ATP recycling and the maintenance of cellular biosynthesis during nutrient deprivation or metabolic stress [116]. Tumor cells are often under metabolic stress owing to hypoxia and nutrient deprivation, and autophagy can help maintain essential cellular functions through molecular recycling. Hypoxia-inducible transcription factor 1-alpha (HIF1-alpha), which promotes autophagy and angiogenesis, is increased in cancer tissues [117]. The “reverse Warburg effect” is a proposed model in which epithelial cancer cells induce the Warburg effect (aerobic glycolysis) in adjacent stromal fibroblasts, which in turn provide lactate and pyruvate for oxidative mitochondrial metabolism in nearby cancer cells [118]. A proteomic analysis of Cav-1-deficient fibroblasts (a characteristic of CAFs, as noted above) revealed an increased transcription level of myofibroblast markers and glycolytic enzymes under normoxic conditions. This was verified in a study of human breast cancer tissues [118]. Subsequent study showed that loss of Cav-1 in mesenchymal stromal cells led to increased aerobic glycolysis and inflammation in the tumor stromal microenvironment via activation of HIF and NFkB [119]. Cav-1 is a negative regulator of nitric oxide (NO), and loss of Cav-1 results in overproduction of NO, which can directly drive mitochondrial dysfunction [115]. Consistent with this, Cav-1-deficient mice treated with 2-deoxy-glucose (glycolysis inhibitor) and metformin (a mitochondrial complex I inhibitor) were extremely sensitive to dual blockade compared to Cav-1 wild-type mice [119]. In another study, when stromal fibroblasts were co-cultured with MCF-7 breast cancer cells, fibroblast autophagy, NO production and mitochondrial dysfunction and reactive oxygen species were increased [120]. This in turn promoted aneuploidy in co-cultured MCF-7 breast cancer cells, which was reversed by antioxidant (*N*-acetyl-cysteine, metformin and quercetin) or NO inhibitor. Finally, TGF-β induced Cav-1 downregulation in CAFs, promoting a fibroblast shift toward catabolic metabolism and promoting the mitochondrial activity of adjacent cancer cells [121].

## 7. Breast Cancer-Associated Fibroblasts Are Involved in Resistance to Breast Cancer Therapy

Breast cancer is the most commonly diagnosed cancer in women worldwide. Treatment for breast cancer, like many cancers, often involves a combination of surgical, radiation and medical therapies. There are multiple options for medical treatments in breast cancer, depending on immunophenotype. These include hormonal therapies targeting the estrogen pathway (such as tamoxifen, aromatase inhibitors and fulvestrant), chemotherapeutic drugs (such as anthracyclines, taxanes, alkylating and platinum agents) and targeted therapies (including agents targeting HER2, mTOR, cyclin-dependent kinases and DNA repair pathways). The combination of early screening and evolving therapies has led to significant improvement in breast cancer outcomes, but drug resistance remains a major obstacle in relapsed and metastatic breast cancer.

Emerging studies suggest that breast cancer-associated fibroblasts may contribute to therapy resistance. *In vitro* studies have demonstrated that breast cancer-associated fibroblasts can modulate tamoxifen resistance in breast cancers via activation of the PI3K/AKT and MAPK/ERK pathways and phosphorylation of estrogen receptor–α at serine 118 [122,123]. Recent work showed that tamoxifen activates the estrogen (G-protein-coupled) receptor (GPER) on breast cancer-associated fibroblasts, promoting proliferation and cell cycle progression through the GPER/EGFR/ERK axis. Moreover, tamoxifen induced CYP19A1 gene expression and estrogen production in breast cancer-associated fibroblasts [124]. As noted above, previous studies have shown that cancer cells induce aerobic glycolysis in adjacent stromal fibroblasts through oxidative stress, driving autophagy. In return, cancer-associated fibroblasts provide a steady supply of nutrients to associated cancer cells [125]. Co-culture of fibroblasts with MCF7 breast cancer cells induced resistance to tamoxifen and fulvestrant, which was also seen in MCF 7 cells treated with mitochondrial fuels, such as l-lactate or ketone bodies [125]. Pharmacologic suppression of MCF7 mitochondria using metformin or arsenic trioxide abrogated this drug resistance. This was accompanied by increased glucose uptake, reflecting metabolic uncoupling between the epithelial cancer cells and fibroblasts. Another mechanism of tamoxifen resistance is through tamoxifen-induced TIGAR (TP53-induced glycolysis and apoptosis regulator), a p53-regulated gene that simultaneously inhibits glycolysis, autophagy and apoptosis and reduces ROS generation, thereby promoting oxidative mitochondrial metabolism. TIGAR overexpression protected MCF7 from tamoxifen-induced apoptosis [125]. A different study showed that cancer-associated fibroblasts induce HMGB1 (high mobility group box 1), a chromatin-associated nuclear protein released from dying tumor cells that has been suggested to mediate cancer progression and resistance to doxorubicin [126]. In MCF7 and T47D breast cancer cells, c-Met upregulation accompanied resistance to fulvestrant, facilitating stimulation by HGF/SF-secreting stromal fibroblasts [127]. Triple-negative breast cancer cells overexpress EGFR (epidermal growth factor receptor), but EGFR inhibitors are not clinically effective [128,129]. Fibroblast HGF activates Met in triple-negative breast cells, which mediates the survival of the cancer cells in the presence of the EGFR inhibitor gefitinib [130]. However, another study demonstrated that cancer-associated fibroblasts enhanced cytotoxic effects of tyrosine kinase inhibitors on human breast cancer cells in 3D culture models [131], suggesting that the questions of whether and how breast CAFs modulate resistance to therapy is an area that merits significant further investigation.

## 8. Conclusions

Breast cancer-associated fibroblasts are the most prevalent cellular component of the breast tumor microenvironment, with the potential for wide ranging functions in breast cancer progression, including cancer initiation, metastasis, angiogenesis, lymphangiogenesis, metabolic reprogramming and therapy resistance. Despite this, fundamental understanding of the critical characteristics of breast cancer-associated fibroblasts, including their origin, definition and biologic heterogeneity, remains to be elucidated. Further investigation into this critical component of the breast cancer microenvironment is likely to yield important insights into the complex connections between cancer cells and their stromal partners, ultimately enabling new therapies against advanced breast cancers.
